# Anthelmintic Efficacy of Strongyle Nematodes to Ivermectin and Fenbendazole on Working Donkeys (*Equus asinus*) in and around Hosaena Town, Southern Ethiopia

**DOI:** 10.1155/2020/4868797

**Published:** 2020-09-24

**Authors:** Haben Fesseha, Mesfin Mathewos, Friat Kidanemariam

**Affiliations:** ^1^Wolaita Sodo University, School of Veterinary Medicine, Department of Veterinary Surgery and Diagnostic Imaging, P.O. Box 138, Wolaita Sodo, Ethiopia; ^2^Wolaita Sodo University, School of Veterinary Medicine, Department of Veterinary Pathology, P.O. Box 138, Wolaita Sodo, Ethiopia; ^3^Mekelle University College of Veterinary Science, Department of Tropical Veterinary Medicine, P.O. Box 2084, Mekelle, Ethiopia

## Abstract

**Background:**

Gastrointestinal helminth parasite infection is a major influencing factor against profitability of working equines all over the world.

**Objectives:**

A study was conducted from October 2016 to May 2017 in and around Hosaena to determine the efficacy of benzimidazole (BZ) and avermectin (AVM) chemical groups against strongyle nematodes in working donkeys.

**Methods:**

A total of 230 donkeys from Hosaena, Soro, Anlemo, and Gombora were randomly allocated into 5 groups of 46 donkeys in each group. All groups, except group 1 (control), were treated with ivermectin 1%, ivertong 10%, fenbendazole, and Fenacure 750 mg, respectively. Fecal samples were collected perrectally before treatment (day 0) and after treatment (day 14), and an egg per gram (EPG) value of >200 was used as a cutoff for inclusion to assess the efficacy of anthelmintics.

**Results:**

Accordingly, the study revealed that AVM was effective against strongyle nematodes of donkeys with the mean fecal egg count reduction (FECR) of 100% in three study areas and 97.2% in one study area, respectively, whereas BZ resistance was suspected in the areas where the drug was tested, with the mean FECR of less than 94% in the three study areas. The study also revealed that 73% of the donkeys were affected with a severe degree of strongyle infection as determined by EPG, while 10.4% of donkeys were affected with a mild degree of *Parascaris equorum* infection.

**Conclusions:**

The findings of the present study are expected to serve as baseline data for future investigations and control actions to design realistic control programs to minimize factors that favor emergence of anthelmintic resistance and improve the overall health of the donkeys. Thus, further detailed studies are needed to determine the factors that reduce anthelmintic efficacy and increase anthelmintic resistance in donkeys.

## 1. Introduction

Helminth parasite infection especially gastrointestinal helminth parasite infection is a major influencing factor against profitable animal production especially equine all over the world [1]. An apparently healthy donkey can harbor over half a million gastrointestinal parasites such as protozoans, trematodes, cestodes, and nematodes. This is because the gastrointestinal tract provides a suitable environment for the survival and proliferation of many of these parasites [2–4]. Moreover, equids including donkeys, horses, and ponies are hosts to a large population of helminths belonging to 28 genera and 75 species of nematodes, 2 genera and 5 species of trematodes as well as 3 genera and 24 species of cestodes [5, 6].

Strategic parasitic control is necessary for the health of donkeys. There are several methods to control different internal parasites of donkeys, and the use of antiparasitic drugs is a widespread practice and has been the major form of control of internal and external parasites [7]. Among these drugs, phenothiazine, piperazine, organophosphates, benzimidazoles, and probenzimidazoles, imidothiazoles (levamisole), tetrahydropyrimidines (pyrantel), and avermectins are the major class of antihelmintic group used in most parts of the world. Benzimidazoles and probenzimidazoles interfere with energy metabolism within the parasite; imidothiazoles, tetrahydropyrimidines, piperazine, organophosphates, and avermectins interfere with parasites' neuromuscular coordination [8–10].

Several types of anthelmintics with different modes of action are available in the market for the control of helminthosis. Nevertheless, the prevalence of anthelmintic-resistant intestinal parasites is a rapidly growing problem in the equine industry [8]. Ivermectin is a broad-spectrum anthelmintic drug which is an analogue of avermectin that belongs to a family of 16-membered macrocyclic lactones and known to increase membrane permeability to chloride ions, possibly as a result of their interaction with chloride ion channels. Besides, it has a wide safety margin that has made it the drug of choice for nematode and arthropod parasitism in cattle, sheep, goats, swine, donkeys, and horses [9].

In equine, according to different published reports, there is an increase in the resistance of strongyles, especially of those belonging to the subfamily Cyathostominae, which is a worldwide phenomenon. They are resistant to the benzimidazole derivatives (thiabendazole, mebendazole, cambendazole, fenbendazole, and oxfendazole), probenzimidazoles, and tetrahydropyrimidines (pyrantel embonate) [1, 11]. Ivermectin has been used for more than 20 years in equids, but up to now, there are no data about the appearance of resistance to this product in equid worms [9, 12]. Ivermectin has a high efficacy (>90% efficacy against adult strongyles and migrating larvae), and a residual effect is not seen with the benzimidazoles and pyrantel embonate [2, 13, 14].

Anthelmintic resistance develops when the parasites survive treatment and pass this ability of resistance to their offspring, resulting in the contamination of the premises with resistant strains. As this continues, the number of resistant worms increases and eventually the product is no longer useful in the treatment of these parasites. The use of anthelmintics and the biology of the parasite are two important factors that influence parasite selection for resistance. Anthelmintic usage includes dosage and route of administration, frequency of treatment, and mechanism of action. The frequency of treatment is regarded as the main factor in selection for resistance. The more frequently a compound is used, the more likely resistance may occur [9, 15].

There are various methods to determine parasite load and identification in the horse industry [16]. Fecal egg counts (FEC) and fecal egg reduction counts (FECR) are considered the simplest and least expensive options to determine the parasite load and how effectively the anthelmintic class being used is reducing the infestation. Several studies have suggested that the fecal egg count reduction test (FECRT) is the gold standard in *in vivo* screening tests to detect anthelmintic resistance [1, 16, 17].

The prevalence and impact of gastrointestinal parasites especially strongyle parasites have been studied in many parts of our country [1, 18]. However, the problem of anthelmintic resistance of strongyle parasites in donkeys has not been investigated yet. Moreover, there are limited data about its fecal egg count (FEC) reduction in donkeys following parenteral administration. Consequently, this study was conducted to evaluate the efficacy of two commonly used chemical groups of anthelmintics (benzimidazoles (BZ) and avermectins (AVM)) against gastrointestinal parasites of donkeys in Hosaena town, southern Ethiopia.

## 2. Materials and Methods

### 2.1. Study Area

The study was conducted from October 2016 to May 2017 in and around Hosaena, the capital of Hadiya zone in Southern Nations, Nationalities, and People's Regional State, Ethiopia. Hosaena is the administrative center of the Hadiya zone at a distance of 232 km away from Addis Ababa and 168 km away from Hawassa, the regional capital. It has a latitude and longitude of 7°33′N and 37°51′E, respectively, with an elevation of 2177 meters above sea level. The mean annual temperature and rainfall are 16.9°C and 1071 mm, respectively. The area exhibits a bimodal rainfall system (long and short rainy seasons). The long rainy season extends from June to September, whereas the short rainy season ranges from mid-February to the end of April [7, 19].

### 2.2. Study Animals

The study animals were working donkeys kept by different peasant associations in and around Hosaena, Hadiya zone, Southern Regional State. The study includes donkeys of all age groups, both sexes, and kept under extensive management systems. The age of the selected working donkeys was determined by the dentition pattern as described in [20] and grouped as young (<5 years), adult (5–10 years), and old (>10 years). Body condition scoring (BCS) of the donkeys was estimated based on the guides described by Elisabeth [21].

### 2.3. Study Design and Sampling Techniques

A field experimental study design was conducted to assess the anthelmintic efficacy of two commonly used chemical groups of anthelmintics (benzimidazoles and avermectins) against gastrointestinal parasites of the donkey in Hosaena town. For this purpose, all fecal specimens were collected from the donkeys and subjected to flotation analysis using the McMaster method [22]. Initially, a total of 230 donkeys were examined for gastrointestinal parasites by using the McMaster technique, and the species of the helminths parasites were determined according to the technique described by Hendrix and Robinson [23]. This is the modified McMaster technique that was also used to determine the eggs of gastrointestinal helminths in horses.

Moreover, the donkeys were classified into different levels based on strongyle egg shedding or egg per gram of feces (EPG) according to the guidelines of Nielsen et al. [24] and Kaplan and Nielsen [25]. Accordingly, the donkeys are categorized into mild if the egg count level is within the range of 0–200 EPG, moderate if the egg count level is 200–500 EPG, and severe if the egg count level is greater than 500 EPG.

Helminth-infected donkeys were treated with four different brands of drugs (ivertong 10%, Fenacure, fenbendazole, and ivermectin) available in the market. Moreover, the donkeys were treated with these four different brands of antihelmintic drugs based on the presence of strongyle eggs in their feces (EPG ≥ 200 eggs/gram of feces) according to the previous work of Kaplan and Nielsen [25].

The doses of each drug were determined according to the manufacturers' recommendations. Then, the donkeys were grouped into five experimental groups (46 donkeys per group). Accordingly, the donkeys in group I were left untreated as a control group, group II donkeys were treated with ivertong 10%, group III donkeys were treated with fenbendazole, group IV donkeys were treated with ivermectin, and group V donkeys were treated with Fenacure. The fecal egg count was determined before (at day 0) and after treatment (at day 14). Thus, the anthelmintic efficacy of the tested drugs was determined using a fecal egg count reduction test (FECRT) [26].

### 2.4. Study Methodology

#### 2.4.1. Sample Collection and Handling

All samples were taken at approximately the same time of the day, that is, in the morning, and processed within 24 hours of collection. Fecal samples were collected directly from the rectum or sometimes from freshly passed feces. Along with sampling, date, name, identification number, age, assigned group, and trial drug were recorded. Then, all fecal samples were transported from the selected site by using iceboxes and analyzed in the regional laboratory.

#### 2.4.2. Anthelmintic Drugs Used in the Study for Assessing Anthelmintic Efficacy

All anthelmintic drugs used in the study were imported into the country by registered companies that are licensed to distribute veterinary drugs. The dosage and route of the application of all drugs used were based on the manufacturers' recommendations (Table 1). Fecal samples were collected again 14 days posttreatment from all donkeys included in the experiment. All the drugs were within their expiry date and stored as per the instructions of the manufacturers.

### 2.5. Data Management and Statistical Analysis

Data collected were stored in Microsoft Excel 2013. Statistical analyses were performed using STATA version 13. The chi-square test was used to compare and measure the association between the prevalence of strongyle infections and age groups. A fecal egg count reduction test (FECRT) was used to determine the anthelmintic efficacy. The resistance of the drugs was tested according to the formula by Coles et al. [26] and World Association for the Advancement of Veterinary Parasitology (WAAVP) recommendations for the detection of anthelmintic resistance in equine and ruminants. Thus, the arithmetic mean of the egg count and nematode burden was calculated by the percentage reduction in mean egg excretion on the 14-day posttreatment.

(1) $$\begin{array}{c} \text{FECR\%}=\frac{\text{pretreatment EPG}-\text{posttreatment EPG}}{\text{pretreatment EPG}}\times 100. \end{array}$$

## 3. Results

### 3.1. Prevalence of Helminths in Donkeys

According to the present study, strongyles were highly prevalent in the donkeys of the study areas. Out of 230, all working donkeys (100%) in the study areas were found to shed nematode eggs while the *Parascaris equorum* (10.4%) is less prevalent in the study areas. Furthermore, the study on the degree of infection of strongyles as indicated by EPG revealed that 73% of the donkeys were with a severe degree of strongyle infection, while there was a 10.4% mild degree of *P. equorum* infection recorded in the donkeys of the study sites (Table 2).

### 3.2. Donkey Age and Fecal Egg Output

**Figure 1:**
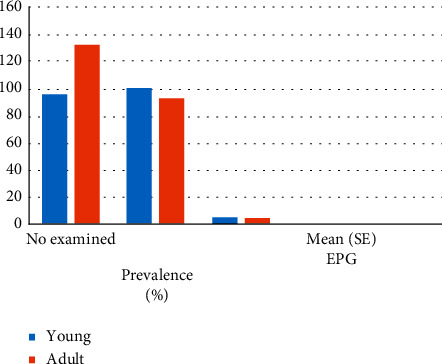
Age-specific egg count of strongyles and *Parascaris*.

In the present study, the association between different age groups and fecal egg output was determined. Out of the total examined, young donkeys (<5 years old) were shedding more strongyles than the adult ones. Besides, there was a statistically significant association (*p* < 0.05) between the average number of eggs per gram of feces (EPG) and the age group of the donkeys (Figure 1).

### 3.3. Efficacy of Anthelmintics against Nematodes of Donkeys

A fecal egg count reduction test was performed in 230 donkeys. The EPG value of >200 was used as a cut-off for inclusion in the study to evaluate the efficacy of four anthelmintics (ivermectin 1%, ivertong 10%, fenbendazole, and Fenacure 750 mg) against strongyles and *Parascaris equorum*, in donkeys from four selected sites (Hosaena, Soro, Anlemo, and Gombora). The mean percentage efficacy of the four drugs in each site surveyed is reported in Table 3 together with the mean EPG value of pre- and posttreatment for each donkey group. Fourteen days posttreatment, ivermectin 1% and ivertong 10% were evaluated to be fully efficacious in all four areas, with the mean FECR of 100% in Soro, Anlemo, and Gombora and 97.2% in Hosaena, respectively, whereas reduced efficacy in fenbendazole and Fenacure 750 mg was suspected in all the areas where the drug was tested, with the mean FECR of less than 95% (Table 3).

## 4. Discussion

In the current study, out of a total of 230 donkeys, 100% of animals were positive for the strongyles which indicate that these nematodes are the most predominant and widespread in the study areas. *Parascaris equorum* (10.4%) was the second most common parasite in the areas. This observation was comparable with the previous report by Hutchison and Mfitlidoze [27] in Australia, where 80% of horses were infected with gastrointestinal parasites. This study has shown that strongyles are highly prevalent in donkeys residing in study areas since all (100%) of the donkeys exceeded the cutoff value (200 EPG). One of the basic principles of selective anthelmintic treatment is a consistency of the relative magnitude of strongyle FECs of individual donkeys over time [6].

One possible explanation for higher EPG values in young donkeys could be that young donkeys generally harbor a greater number of mucosal larval stages than do older individuals, young (61.4%) and adult (38.6%), respectively [5, 28]. The development of an age-related immunity, which results in lower worm burdens in old individuals compared to the young, has been demonstrated in experimental studies using ponies of different ages [12, 29].

The decreased worm fecundity is another manifestation of immunity that could explain the age differences observed in mean EPG values. For example, it has been shown that immunity to strongyles in foals results in a reduction in worm fecundity [30, 31]. An additional reason for higher EPG values in young donkeys could be that the anthelmintic treatments performed previously were less effective in young donkeys compared to adult animals. This suggestion is based on the interactions that were found between anthelmintic treatment and donkey age.

According to the present study, there was a reduction in the efficacy of benzimidazole drugs against the strongyle of donkeys. Similar observations were recently reported by Lind et al. [32] in Sweden and Cernea et al. [33] in Romania. The reduction in efficacy recorded in benzimidazole drugs in the present study could be associated with a low frequency of anthelmintic treatment, poor quality of the drug compared to other anthelmintics, or the animal that develops acquired resistance through time, or the parasite which itself can mutate and develop resistance gene against the drug. The fact that anthelmintics can easily be accessed from illegal sources implies that this risk factor for antihelmintic resistance must be addressed through the expansion of veterinary extension programs. This finding supports the reports of previous studies [12, 31, 34]. This could potentially result in selective perpetuation of resistant isolates of the parasite, consequently posing the future risk of drug resistance as anthelmintic resistance is mostly inherited [1, 35].

In three of the study sites, namely, Hosaena, Soro, and Anlemo, fenbendazole (FBZ) had lower (<95%) fecal egg count reduction percentage which indicated that strongyle nematodes are resistant to fenbendazole. This may be due to frequent usage, species of the parasite, and poor management system. This report is in agreement with a field study conducted by Lind et al. [32] in Sweden and Cernea et al. [33] in Romania; the FBZ-treated groups met the criteria for resistance. However, studies carried out in Italy, the United Kingdom, and Germany by Traversa et al. [17] showed the effectiveness of fenbendazole against horse strongyles (99.49%). They reported that horses have been dewormed once per year or less or never. This may probably be the reason why the resistance against fenbendazole was not found in these reports.

All brands of avermectins had a fecal egg count reduction percentage greater than 95% in all study areas. This is in accordance with the result of Traversa et al. [17] in Italy, the United Kingdom, and Germany, Lyons et al. [36] in the USA, and von Samson-Himmelstjerna et al. [37] in Germany from different horse farms. They reported that reduction in activity of ivermectin and moxidectin against small strongyles seems to be due to the survival of some of the luminal immature stages in the large intestines after treatment. This might be due to the absence of resistance which could also be explained by the low sensitivity of the FECRT to detect levels of resistance below 99% [2, 13]. Nevertheless, it is known that the FECRT is not a very sensitive method for the early diagnosis of anthelmintic resistance, and owners, as well as veterinary practitioners, need to consider how to use effective anthelmintics for donkeys.

Accordingly, based on the analysis, resistance to fenbendazole anthelmintics has been suspected that could be associated with a low frequency of anthelmintic treatment, poor quality of the drug compared to other anthelmintics, or the animal that develops acquired resistance through time, or the parasite which itself can mutate and is able to develop resistance gene against the drug. However, in the present study, based on the analysis, ivermectin was effective in the study areas. This was in contrast with the report of Papadopoulos et al. [38] who explained that fecal egg reduction following ivermectin treatment was reported to be significantly reduced and resistance was not detected in Greece. This was due to the fact that the monitoring activity applied to the efficacy of ivermectin against intestinal strongyles was very high. This difference in reduced efficacy in this study area may be due to drug usage strategies, the biology of the parasite, and the quality of the available anthelmintic drugs.

## 5. Conclusion and Recommendations

Both benzimidazoles (BZ) and avermectins (AVM) were the two commonly used chemical groups of anthelmintic drugs in and around Hosaena town to control the parasitic infestation. The present study showed that strongyles are highly prevalent (100%) in donkeys in Hosaena town, southern Ethiopia. The study reported a reduction in the efficacy of benzimidazoles used to treat nematodes of donkeys of the study areas. However, a single oral dose (14 days apart) of ivermectin drench formulation administered at a dose rate of 10 mg/kg body mass was highly efficient against naturally acquired infections of adult strongyle species and *Parascaris equorum*, nematodes in donkeys. In conclusion, control and preventive treatments with anthelmintics should be more integrated with other measures, such as supplementation of feed and good management, awareness creation to donkey owners by continuous veterinary extension about the importance of wise and smart use of anthelmintics including the effectiveness of these drugs, avoidance of frequent dosing and underdosing, and monitor drugs for resistance every two to three years. Besides, further detailed studies have to be planned and conducted to investigate the extension of this phenomenon in horse parasites covering wider study areas and different horse establishments in Ethiopia.

## Figures and Tables

**Table 1:** Details of anthelmintic drugs used in the FECRT efficacy evaluation.

Trade name	Generic name	Manufacturer	Dose per kg body weight	Route of administration
Granule Febenda	Fenbendazole 20%	China	7.5 mg/kg	With grain feed
Fenacure 750	Fenacure 750 mg	India	10 mg/kg	With grain feed
Ivervic	Ivermectin 1%	China	0.2 mg/kg	Orally
Fangtong	Ivertong 10%	China	0.2 mg/kg	Orally

**Table 2:** Prevalence of helminth infestation in donkeys of the study areas.

Helminths (EPG)	Total examined	Degree of infection (%)			95% CI	Overall proportion
		Mild	Moderate	Severe		
Strongyle egg	230	7 (3.04)	55 (23.9)	168 (73.04)	2.49–2.68	100
*P. equorum* egg	—	24 (10.4)	0	0	0.12–0.32	10.4

**Table 3:** Efficacy of the tested anthelmintic drugs by fecal egg count reduction (FECR).

Study site	Treatment group	Number of animals	Mean EPG		FECR (%)	Status of resistance
			Before treatment	After treatment		
Hosaena	Ivertong 10%	11	1063.636	0	100	Very susceptible
	Ivermectin	12	991.7	25	97.5	Susceptible
	Fenbendazole 20%	11	1350	142.8	89.4	Resistant
	Fenacure 750 mg	12	1212.5	112.5	90.7	Resistant

Soro	Ivertong 10%	12	16900	300	98.22	Susceptible
	Ivermectin	11	14000	0	100	Very susceptible
	Fenbendazole 20%	11	8900	1100	87.6	Resistant
	Fenacure 750 mg	12	16200	1300	91.9	Resistant

Anlemo	Ivertong 10%	11	27400	400	98.5	Very susceptible
	Ivermectin	12	15900	0	100	Susceptible
	Fenbendazole 20%	12	27100	3300	87.8	Resistant
	Fenacure 750 mg	11	18200	1000	94.5	Resistant

Gombora	Ivertong 10%	12	16200	400	97.5	Susceptible
	Ivermectin	11	14500	0	100	Very susceptible
	Fenbendazole 20%	12	13300	0	100	Very susceptible
	Fenacure 750 mg	11	14100	0	100	Very susceptible

## Data Availability

The data used to support the findings of this study are available from the corresponding author upon request.
